# A georeferenced rRNA amplicon database of aquatic microbiomes from South America

**DOI:** 10.1038/s41597-022-01665-z

**Published:** 2022-09-13

**Authors:** Sebastian Metz, Paula Huber, Erick Mateus-Barros, Pedro C. Junger, Michaela de Melo, Inessa Lacativa Bagatini, Irina Izaguirre, Mariana Câmara dos Reis, Maria E. Llames, Victoria Accattatis, María Victoria Quiroga, Melina Devercelli, María Romina Schiaffino, Juan Pablo Niño-García, Marcela Bastidas Navarro, Beatriz Modenutti, Helena Vieira, Martin Saraceno, Carmen Alejandra Sabio y García, Emiliano Pereira, Alvaro González-Revello, Claudia Piccini, Fernando Unrein, Cecilia Alonso, Hugo Sarmento

**Affiliations:** 1 Laboratorio de Ecología Acuática, Instituto Tecnológico de Chascomús (INTECH), UNSAM-CONICET, Av. Intendente Marino Km 8.200, (7130) Chascomús, Buenos Aires, Argentina; 2 Sorbonne Université, CNRS, UMR7144 Adaptation et Diversité en Milieu Marin, Ecology of Marine Plankton (ECOMAP), Station Biologique de Roscoff SBR, 29680 Roscoff, France; 3 Laboratorio de Plancton Instituto Nacional de Limnología (INALI), CONICET-UNL, Ciudad Universitaria, Paraje El Pozo, C. P, 3000 Santa Fe, Argentina; 4 Laboratory of Microbial Processes & Biodiversity, Departamento de Hydrobiologia, Universidade Federal de São Carlos (UFSCar). Rodovia Washington Luiz, São Carlos, São Paulo 13565-905 Brazil; 5 University of Quebec at Montreal, Department of Biological Science. Centre-Ville, C.P, 8888 Montreal, (Quebec) Canada; 6 Laboratório de Ficologia, Departamento de Botânica, Universidade Federal de São Carlos (UFSCar). Rodovia Washington Luiz, São Carlos, São Paulo 13565-905 Brazil; 7 Depto. de Ecología, Genética y Evolución, IEGEBA (UBA-CONICET), Facultad de Ciencias Exactas y Naturales, Universidad de Buenos Aires (UBA). Intendente Güiraldes 2160, C1428EHA Buenos Aires, Argentina; 8 Sorbonne Université/Centre National de la Recherche Scientifique, UMR 7144, Adaptation et Diversité en Milieu Marin, Station Biologique de Roscoff, Place Georges Teissier, Roscoff, France; 9 Departamento de Ciencias Básicas y Experimentales, Universidad Nacional del Noroeste de la Provincia de Buenos Aires (UNNOBA), Roque Sáenz Peña 456, 6000 Junín, Argentina; 10 Centro de Investigaciones y Transferencia del Noroeste de la Provincia de Buenos Aires (CITNOBA) – UNNOBA-UNSAdA-CONICET, Monteagudo 2772, 2700 Buenos Aires, Argentina; 11 Escuela de Microbiología, Universidad de Antioquia, Cl. 67 ##53-108, Medellín, Antioquia Colombia; 12 Instituto de Investigaciones en Biodiversidad y Medio Ambiente (INIBIOMA), CONICET-Universidad Nacional del Comahue, Quintral 1250, R8400 San Carlos de Bariloche, Río Negro Argentina; 13 Czech Academy of Sciences, Biology Centre, Hydrobiology Institute, Na Sádkách 702/7, 370 05 Ceske Budejovice, Czechia; 14 Centro Universitario Regional del Este. Universidad de la República, Ruta nacional N° 9 intersección con ruta N°15, CP 270000 Rocha, Uruguay; 15 Departamento de Ciencia y Tecnología de los Alimentos, Facultad de Veterinaria, UDELAR. Alberto Lasplaces 1620, CP 11600 Montevideo, Uruguay; 16 Departamento de Genómica, Instituto de Investigaciones Biológicas Clemente Estable, MEC. Av. Italia 3318, 11600 Montevideo, Departamento de Montevideo Uruguay; 17 Laboratorio de Ecología Microbiana Acuática, Departamento de Microbiología, Instituto de Investigaciones Biológicas Clemente Estable, MEC. Av. Italia 3318, 11600 Montevideo, Departamento de Montevideo Uruguay

**Keywords:** Microbial ecology, Molecular ecology

## Abstract

The biogeography of bacterial communities is a key topic in Microbial Ecology. Regarding continental water, most studies are carried out in the northern hemisphere, leaving a gap on microorganism’s diversity patterns on a global scale. South America harbours approximately one third of the world’s total freshwater resources, and is one of these understudied regions. To fill this gap, we compiled 16S rRNA amplicon sequencing data of microbial communities across South America continental water ecosystems, presenting the first database **µSudAqua[db]**. The database contains over 866 georeferenced samples from 9 different ecoregions with contextual environmental information. For its integration and validation we constructed a curated database (**µSudAqua[db.sp]**) using samples sequenced by Illumina MiSeq platform with commonly used prokaryote universal primers. This comprised ~60% of the total georeferenced samples of the **µSudAqua[db]**. This compilation was carried out in the scope of the µSudAqua collaborative network and represents one of the most complete databases of continental water microbial communities from South America.

## Abstract (summary)

**Table:** 

Measurement(s)	taxonomic diversity assessment by targeted gene survey
Technology Type(s)	next generation DNA sequencing
Sample Characteristic - Organism	Bacteria • Archaea
Sample Characteristic - Environment	aquatic environment
Sample Characteristic - Location	South America

## Background & Summary

Microorganisms are the main drivers of biogeochemical cycles in freshwater ecosystems^1–4^. Due to their high abundances and activities and to their collective metabolic and phylogenetic diversity, prokaryotes support aquatic food webs and regulate the magnitude and recycling rates of major elements^5^. Thus, understanding the microbial diversity patterns is a fundamental topic in modern Microbial Ecology and a key step for advancing our knowledge on bacterial-mediated processes across continental water ecosystems.

**Fig. 1:**
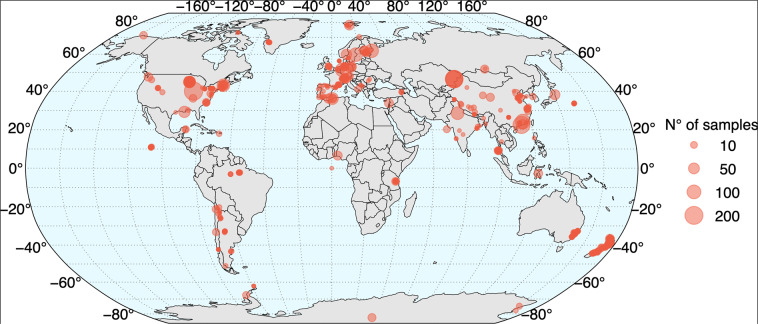
Global distribution of amplicon sequencing samples from continental water systems using HTS. The information was acquired from MGnify (https://www.ebi.ac.uk/metagenomics/) resource by searching for non-marine aquatic samples, obtained by amplicon or metabarcoding experimental types. The geographical coordinates were retrieved for 4,691 samples from a total of 7,832 using the metadata available from each sample.

Despite the extensive application of amplicon sequencing by high-throughput technologies (HTS), there are still important gaps in the study of aquatic microbial diversity^6–8^. For example, a rough mapping of the worldwide distribution of amplicon sequencing studies (Fig. 1), clearly shows that most of them are from the northern hemisphere, particularly from Europe and the United States – Canada, while the Southern Hemisphere has a contrasting underrepresentation^9,10^. This is especially true in South America and Africa, where sequencing studies are still scarce and generated from isolated efforts.

The Southern Hemisphere covers a comparatively high share of the surface and volume of the continental and marine ecosystems in the world. In particular, South America is considered the “continent of water”, harboring 6 out of the 10 largest rivers in the world in terms of water discharge, draining about 30% of the continental freshwater that reaches the ocean^11^. This water flows through five huge hydrological river basins: the Amazonas (6,000,000 km^2^), Del Plata-Paraná/Paraguay (2,600,000 km^2^), Orinoco (990,000 km^2^), Araguaia-Tocantins (757,000 km^2^), and São Francisco (634,000 km^2^)^12,13^. In addition, a great number and diversity of lentic water bodies are also prominent features that tend to occur in lake districts and wetlands as a result of the main climatic and geomorphological processes acting on regional scales^14^.

Furthermore, the South American continent comprises a large ecological heterogeneity^15,16^. South America covers about 15% of the global land area (17,870,218 km^2^) and spans a broad latitudinal range, extending from 12° 28′N (Punta Gallinas, Colombia) to 55° 59′S (Cabo de Hornos, Chile). According to the biogeographic regionalization by Cabrera & Willink^15^, South America belongs to the Neotropical region, except the southernmost area, which is assigned to the Antarctic region. Owing to the wide latitudinal coverage, a large variety of climates occur, with much of the continental mass located within the intertropical belt, large regions of Chile, Argentina, and Uruguay laying in the Southern Temperate Zone, and the southern tip of the continent extending into sub-Antarctic latitudes. Due to the high habitat heterogeneity and a complex geological history, South America is considered a hotspot of biodiversity, being the most species-rich region on Earth^17,18^. This fact, added to its location in a predominantly maritime hemisphere, offers a unique opportunity for comparing empirical patterns found originally for Northern Hemisphere aquatic ecosystems. Pioneering studies have shown a geographically biased picture of the aquatic microbiome, leading to overlook differences in structure and functioning of microbial communities^19^. That limits, for instance, our understanding of the expected scenarios resulting from the current pressures experienced by the aquatic ecosystems^20^.

In order to reduce this gap, we performed an exhaustive bibliographic search, collected and annotated data of bacterial communities from South American continental waters. We constructed the first 16S rRNA amplicon sequencing database of South America, the **µSudAqua[db]** containing 866 georeferenced samples. For its integration in further works, preventing biases by the sets of primers and sequencing methodology used, we constructed a curated database (**µSudAqua[db.sp]**) which contains over 509 samples of the V3-V4 region of the 16S rRNA gene sequenced by the Illumina MiSeq technology with the commonly used primers proposed by Herlemann & collaborators^21^.

This work is a result of the µSudAqua collaborative network, a Latin American network in Aquatic Microbial Ecology. This network emerged as an initiative to nucleate researchers of the field to join efforts in consolidating a regional critical mass. Its main objectives are to strengthen and expand the interactions between aquatic microbial ecologists, to contribute to the development of a community feeling at the regional level, and to provide a fruitful space for long-term collaboration in research and training of human resources. More information can be found at the µSudAqua website (https://microsudaqua.netlify.app/).

## Methods

### Data compilation

The **µSudAqua[db]** database was constructed with samples from published papers and new data generated in the scope of this work (Table 1). We only considered those studies fitting with the following criteria: 1) samples were obtained from continental water systems of South America; 2) the whole bacterial community was studied using high-throughput amplicon sequencing of the 16S rRNA gene; 3) the 16S rRNA gene was subject to amplification using universal primers (i.e. studies using group-specific primers or functional genes were not included); 4) sequencing data were publicly available or provided by the authors of the study upon request; 5) the samples could be georeferenced.

**Table 1:** Data sources of the samples used to build the **µSudAqua[db]**.

Ecoregion	Sub-ecoregion	Sample number	References
18. Central Andes	18.1 Central High Andes/Puna	48	^74–78^
19. Southern Andes	19.2 Valdivian Forested Hills and Mountains	12	^79,80^
20. Amazonian-Orinocan Lowland	20.4 Amazon and Coastal Lowlands	173	^81–85^
21. Eastern Highlands	21.2 Cerrados	120	^86–97^
21. Eastern Highlands	21.4 Atlantic Forests	207	
22 Grand Chaco	22.2 Humid Chaco	59	^98^
23. Pampas	23.1 Northern Rolling	37	^99,100^
23. Pampas	23.2 Southern Flat Pampas	127	^101–104^
24. Monte-Patagonian	24.2 Patagonian Tablelands	83	^105–107^

Sample metadata were collected from the published papers or provided by the authors of the current work. The altitude was automatically extracted based on the sampling location, using the QGIS geographic information system software (https://qgis.org/). Each sample was assigned to an environmental type (e.g. shallow and deep lakes, rivers, streams, reservoir, swamps) and an ecoregion (section Ecoregions description). Besides, the georeferenced location and procedures adopted for the sampling and sequencing were fully recovered. The complete list of metadata recovered and its description is presented in Table 2. The samples information used to build the database is available as an accessed as plaintext (TSV format) at Zenodo repository^22^.

**Table 2:** Metadata associated to the samples used to build the **µSudAqua[db]** database. Each sample is identified by an sample indentifier and the corresponding Run accession number from the GenBank. Moreover, each sample was assigned to a geographical location, ecoregion, sub-ecoregion and environment.

Metadata	Description
**SampleID**	Sample identify
**SamplingLocation**	Location identify
**Country**	Country the observations belongs to
**Ecoregion**	Ecoregion Name
**SubEcoregion**	Sub-Ecoregion Name
**EnvironmentName**	Type of habitat the sample was taken from
**SystemType**	Type of system the sample was taken from
**Lat**	Geographic Latitude in decimal degree
**Long**	Geographic Longitude in decimal degree
**Altitude**	Altitude of sampling location in meters above sea level [m.a.s.l]
**Depth**	Sample depth in meters [m]
**CollectionDate**	Date of the sampling event
**SizeFraction**	Size fraction (µm) upper and lower threshold
**FilterPreservation**	Solution in which the filter was preservated
**StorageTemperature**	Temperature at which sample was stored C
**NucleicAcidType**	Nucleic acid target (RNA/DNA)
**ExtractionMethod**	Method used for the nucleic acid extraction
**MechDisruptionMethod**	Method used to disrupte the cells
**StorageDuration**	Duration for which sample was stored
**SeqPlatformName**	Next-generation sequencing plataform which the reads were generated
**SeqPlatformModel**	Next-generation sequencing plataform model which the reads were generated
**LibraryLayout**	If single or paired end reads method was used
**LibraryStrategy**	Sequencing technique implemented for the library
**LibrarySource**	Type of source material that is being sequenced.
**LibrarySelection**	Method used to select and/or enrich the material being sequenced
**SSUgeneName**	Name of the target gene
**HypervariableRegion**	Hypervariable region of 18 S/16S gene target
**FWD_PimerName**	Forward primer name
**FWD_PrimerSequence**	Sequence associated wite the forward primer (5′-3′)
**REV_PrimerName**	Reverse primer name
**REV_PrimerSequence**	Sequence associated with the reverse primer (5′-3′)
**PrimerReference**	Bibliographic citation associated Primer used
**Reference**	Bibliographic citation associated with the data
**Database**	Public database hosted the raw sample
**StudyAcc**	ENA Study Accession Number
**SampleAcc**	ENA Sample Accession Number
**ExperimentAcc**	ENA Experiment Accession Number
**RunAcc**	ENA Run Accession Number
**ReadCount**	Total number of sequenced reads
**BaseCount**	Total number of sequenced nucleotides
**Included_in_usudaquadb_sp**	If the sample is included in the µSudAqua[db.sp] or not
**NumReads_initial**	Number of reads in the sample fastq
**NumReads_final**	Total number of high quality reads after read trimming, read length filtering, and removal chimeras
**NumbASVs**	Total number of Amplicon Sequences Variants (ASVs) defined by DADA2 after removal chimeras
**RevisionDate**	Date of revision and information update
**Download_ftp_R1**	Ftp link to download the forward raw fastq
**Download_ftp_R2**	Ftp link to download the reverse raw fastq

Technical information regarding of sampling procedure, nucleic acid extraction methodologies and sequencing strategies are also described. For samples from the **µSudAqua[db.sp]** database the number of high quality reads and Amplicon Sequences Variants (ASVs) defined is also indicated.

The **µSudAqua[db]** was used as a seed to construct the curated database, **µSudAqua[db.sp]**, which contains a subset those samples sequenced with 1) Illumina MiSeq technology and; 2) the commonly used set of primers proposed by Herlemann & collaborators^21^.

The microbial communities were obtained with different filtration strategies. In some environments, water samples were pre-filtered to exclude larger particles, or to split the microbial community in free-living and particle attached fractions (Table 3). Even though different DNA-extraction methods were used (Table 3), the V3-V4 regions of the 16S rRNA gene were amplified using the same set of bacterial universal primers 341 F (5′-CCTACGGGNGGCWGCAG-3′) and 805 R (5′-GACTACHVGGGTATCTAATCC-3′)^21^. Samples of each project were indexed with Nextera XT v2 kit, and sequenced using the Illumina MiSeq technology in different sequencing facilities. The samples were obtained mostly from surface waters (0–50 cm) of continental systems with different limnological characteristics and different spatial and temporal coverage through six ecoregions (Table 3).

**Table 3:** **µSudAqua[db.sp]** database sample description by ecoregions.

Eco-region	Sub eco-region	Country	Type of system	Latitude	Longitude	Size fraction	Extraction method (N° of samples)	N° of samples
20. Amazonian-Orinocan Lowland	20.4 Amazon and Coastal Lowlands	Brazil	Floodplain lake	2°06′S–2°16′S	55°13′W–55°48′W	0.2–1.2 µm 0.2–1.2 µm > 3 µm > 3 µm	Epicentre MGD08420 (3) Phenol-Chloroform (9) Epicentre MGD08420 (4) Phenol-Chloroform (8)	24
20. Amazonian-Orinocan Lowland	20.4 Amazon and Coastal Lowlands	Brazil	Rivers and floodplain lakes	3°49′S	60°18′W	0.22–3 µm > 3 µm	Phenol-Chloroform (33) Phenol-Chloroform (36)	69
21. Eastern Highlands	21.4 Atlantic Forests	Brazil	Shallow lakes	19°58′S–24°36 S	44°21′W–52°19′W	0.22–1.2 µm 0.22–1.2 µm	Phenol-Chloroform (53) PowerSoil extraction kit (11)	64
21. Eastern Highlands	21.2 Cerrados	Brazil	Shallow lakes	19°59′S–23°52′S	47°10′–51°05′W	0.22–1.2 µm 0.22–1.2 µm	Phenol-Chloroform (17) PowerSoil extraction kit (5)	22
21. Eastern Highlands	21.2 Cerrados	Brazil	Reservoirs	22°10′S	47°54′W	>0.22 µm	Qiagen Dneasy Power Water	15
21. Eastern Highlands	21.2 Cerrados	Brazil	Reservoirs	20°40′S–22°31′S	48°31′W–51°16′W	0.22–3 µm > 3 µm	PowerSoil extraction kit (24) PowerSoil extraction kit (24)	48
22 Grand Chaco	22.2 Humid Chaco	Argentina	Rivers and floodplain lakes	31°37′S–31°50′S	60°28′W–60°48′W	0.22–50 µm	Phenol-Chloroform	59
23. Pampas	23.2 Southern Flat Pampas	Argentina	Shallow lakes	34°28′S–38°55′S	56°58′W–63°05′W	0.22–45 µm	CTAB-Chloroform-Isoamyl alcohol	50
23. Pampas	23.2 Southern Flat Pampas	Argentina	Urban streams	34°41′S–34°51′S	58°18′W–58°21′W	0.22–3 µm	CTAB-Chloroform-Isoamyl alcohol	14
23. Pampas	23.2 Southern Flat Pampas	Argentina	Shallow lakes	34°34′S–35°51′S	57°52′W–61°03′W	0.22–54 µm 0.22–45 µm	CTAB-Chloroform-Isoamyl alcohol (53) CTAB-Chloroform-Isoamyl alcohol (10)	63
19. Southern Andes	19.2 Valdivian Forested Hills and Mountains	Argentina	Deep and shallow lakes	41°00′S–41°21′S	71°18′W–71°49′W	>0.22 µm	PowerSoil extraction kit	7
24. Monte-Patagonian	24.2 Patagonian Tablelands	Argentina	Rivers and reservoirs	39°15′S–40°45′S	68°44′W–71°06′W	0.22–18 µm	CTAB-Chloroform-Isoamyl alcohol	15
24. Monte-Patagonian	24.2 Patagonian Tablelands	Argentina	Shallow lakes	46°71′S–47°09′S	71°03′W–71°19′W	0.22–3 µm	CTAB-Chloroform-Isoamyl alcohol	11
24. Monte-Patagonian	24.2 Patagonian Tablelands	Argentina	Shallow lakes	46°44′S–48°40′S	71°03 W–71°31′W	0.22–3 µm	CTAB-Chloroform-Isoamyl alcohol	41

In all cases the V3-V4 region of gen 16S rRNA was sequenced with illumina MiSeq using the primers 341 F (5′-CCTACGGGNGGCWGCAG-3′) and 805 R (5′-GACTACHVGGGTATCTAATCC-3′).

Amplicon sequences from the **µSudAqua[db.sp]** were processed using DADA2 v1.10.0^23^, after primers trimming by Cutadapt v1.18^24^. Each sequencing project was analyzed separately with the same filtering parameters as recommended by Callahan & collaborators^23^. The quality of the samples was explored using the functions *fastx_eestat* and *fastx_info* from USEARCH v10.0.240^25^ to define the filtering parameters. This was then performed using the *filterAndTrim* function from DADA2 with the following quality values: maxEE = c(2,2) and truncLen = c(250,220). Only samples with more than 10,000 reads were analyzed.

To increase sensitivity to rare variants and avoid chimeras and sequencing errors, we used the “pool” option from *dada* function. The chimera sequences were excluded after merging the different projects using the functions *removeBimeraDenovo* and *mergeSequenceTables*, respectively. The taxonomic classification was performed using BLAST v2.5^26^ with the blastn algorithm (e-value = 0.0001) and the SILVA database (SSU Ref 132 NR 99^27^) as a reference. The Amplicon sequence variants (ASVs) were classified into 7 different taxonomic groups. The contribution of each group was calculated as their relative abundance to the total number of reads, and the richness was defined as the total number of ASVs. The scripts used for DADA2 and sample description are available in GitHub (https://github.com/microsudaqua/usudaquadb).

### Ecoregions description

To define the ecoregions, we adopted the level II classification proposed by Griffith & collaborators^28^ for Central, South America and the Caribbean. The characteristics of each ecoregion and subregion are briefly described below.

18. Central Andes

18.1 Central High Andes, Chile

The Central High Andes ecoregion extends from southern Peru, through Chile and Bolivia, to northern Argentina (5.18°–38.44° S, 78.17°–70.24° W). The landscape is typically mountainous, with snow-capped peaks, plateaus and valleys^29^. The ecoregion occupies an area of 140,960 km^2^ and lies within the altitudinal range between 3,200 and 6,600 m^15^. Its climate varies from temperate to cold, with an annual average temperature between below zero and 15 °C. This region is dry, with precipitation between 250 and 500 mm per year^29,30^. It is considered as a transitional zone between the wet puna to the north and west, and the dry puna to the south. This ecoregion has several high-elevation wetlands comprising both fresh and saline lakes, salt flats, temporary endorheic basins, as well as permanent rivers and streams fed by snowmelt. They regulate water flow by retaining water during the wet season and releasing it during the dry season. The salt flats, or salares, represent remnants of extensive paleolakes^29^.

19. Southern Andes

19.2 Valdivian Forest Hill and Mountains

The Valdivian Temperate Forests ecoregion is in the southern cone of South America (33.02°–46.91° S, 70.55°–74.51° W). It covers a narrow continental strip between the western slope of the Andes and the Pacific Ocean (area: 248,100 km^2^). The climate is temperate cool (mean annual temperature is 8.7 °C) with predominance of westerly winds, and annual precipitation of 1,500 mm^31^. The ecoregion is characterized by a profuse hydrographic system including large and deep lakes (mainly glacial origin)^32,33^ and small and shallow lakes^34,35^. The main rivers fed from these Andean waters, run across the plateau steppe and outflow to the Atlantic Ocean, but there are also other rivers that cross the Andes flowing towards the Pacific Ocean. Deep lakes (Zmax > 100 m) have a warm monomictic thermal behavior^36^. Nevertheless, small and shallow lakes (Zmax ~12 m) are dimictic or polymictic^34^. These lakes have very low nutrient (ranging from ultra-oligotrophic to oligotrophic status) and dissolved organic carbon concentrations, and high transparency to different wavelengths, which would imply high exposure to ultraviolet radiation^37–40^.

20. Amazonian-Orinocan Lowland

20.4 Amazon and Coastal Lowlands

The Amazon river basin is the largest in the world, comprising an area over 6 million km^2^, extending from 5°N to 17°S, and 79°W to 46°W. Basin sources are mostly located in the northern region of Brazil, starting in the Andes mountains of Peru and end in the Atlantic Ocean in the Brazilian coast^41^. The climate in the basin is in general hot and humid with mean annual temperature between 24 to 28 °C^42^. The average annual precipitation is ~2,200 mm, ranging from ~3,000 mm in the west to ~1,700 mm over the southeast of the basin^43^. The Amazon basin comprise numerous large rivers, tributaries, and large extensions of floodplains with thousands of lakes and associated wetlands linked to each other^44^. These systems vary from permanent to periodically flooded depending on the hydrological cycle, namely the flood pulse^38^. This flood pulse has a profound effect on the productivity, transport of elements and biotic interactions within these ecosystems^41,45^.

21. Eastern Highlands

21.2 Cerrado

The Cerrado is the second largest ecoregion in South America. It comprises the Brazilian central region (2.05°–23.77° S, 45.29°–54.37° W), and covers an area over 2 million km^2,^ ^46^. It is a savannah domain, characterized by a tropical climate (mean annual temperature average: 22–27 °C), with dry winters and rainy summers^46^. Annual precipitation typically ranges from 1,200 to 1,800 mm and soil is usually acid and nutrient-poor^47^. The Cerrado altitude has little variation, being maximum only in the central highlands, from where important springs come out and end up contributing to form the three largest water basins in South America (Amazon, São Francisco and Del Plata-Paraná/Paraguay)^48^. There are very few natural lakes in this region, and most water bodies are either dammed shallow lakes or large hydroelectric reservoirs. As reservoirs are mainly found near cities, the nutrient inputs, pH and trophic state can vary^49,50^.

21.4 Atlantic Forests

The Atlantic Forests region is mainly located in Brazil, spanning along the Atlantic coast, and extending inland to Argentina and Paraguay (distributed from 5.00° S to 28.00° S and 35.14° to 53.56° W^51^). This ecoregion is a wide tropical (mean annual temperature ~23 °C), humid biome known mainly by its long line of coastal rainforest^51^. The coast is humid all over the year, with an annual precipitation typically ranging from 1,800 to 3,600 mm. This ecoregion is characterized by different formations like deciduous and semi-deciduous continental forests, bogs and mangroves, and grasslands^52^. Landscape can be flat and lentic environments in the countryside are either human made dammed creeks used for cattle ranching and crop irrigation or large hydroelectric reservoirs. Along the Brazilian Atlantic coast, lentic ecosystems are shallow lakes dug into the mountainside, or squeezed into the narrow strip between the mountain chain and the ocean^53^. There are also some herbaceous/shrubby sand-dune ecosystems, called Restinga, that form perennial or temporary coastal shallow lagoons^54^, which encompass wide environmental gradients (e.g.: trophic state, humic substances, salinity) that greatly influence aquatic biodiversity^55^.

22. Gran Chaco

22.2 Humid Chaco

#### Lakes and rivers from the Paraná floodplain system

The Paraná River is the second largest river of South America with a mean annual discharge of ~17,000 m^3^ s^−1^ and a drainage area of 2.6 10^6^ km^2^. The headwaters are fully developed in Brazil and it travels 3,800 km along a main north to south direction through tropical to temperate latitudes up to its mouth in the Río de la Plata Estuary with mean annual temperatures of ~12.5 °C^56^. The middle stretch of the river begins downstream from the confluence with the Paraguay River (Argentina). Climate is humid subtropical, with annual precipitation between 900 to 1,000 mm. At this stretch, the river is characterized by a well-defined main channel and a large floodplain about 20 to 40 km wide, located by its right margin. Thousands of permanent shallow lakes and temporary environments occupy the floodplain which is flooded and drained by a well-developed and relatively stable fluvial network^57^. The system dynamic is subject to hydro-sedimentological pulses that occur with different magnitudes and constitute the main driving factor of the limnological features and the biota^38,58^, particularly, the microbial communities^59–61^.

23. Pampas

23.1 Uruguayan savanna, Uruguay

The ecoregion Uruguayan savanna comprises an area of 355,605 km^2^ which includes the whole country of Uruguay (30°–34° S, 53°–58° W) and extends mostly towards the southern part of Brazil to a small section of the Argentina^62^. The climate of this region is temperate, without dry season, and with hot summer^63^. The mean annual temperature ranges between 16 and 20 °C. The mean annual rainfall lies between 1,100 and 1,400 mm and is highly variable between years. This ecoregion encompasses the outlet of the Río de la Plata basin where a dense fluvial network, along with a series of coastal lagoons and numerous artificial lakes can be found. Rivers and streams are characterized by small slopes and rapid filling and draining^64^. Coastal lagoons, formed due to marine regressions and transgressions in the Holocene, are located at the Atlantic coast^65^ and their size and age increase towards the East. They are characterized by large gradients in salinity, light penetration and nutrient concentrations, and their hydrological cycle strongly determines the composition and activity of the bacterial communities^66,67^.

23.2 Southern Flat Pampas

The Pampa ecoregion extends westward across central Argentina (30.37°–38.98° S, 57.60°–62.31° W), from the Atlantic coast to the Andean foothills^32^. It is an extensive plain area (398,966 km^2^), except for the two, almost parallel, hill systems that cross the area in a NW–SE orientation (Sierras de Tandilia and Sierras de Ventania). The climate of this region is temperate and humid, with mean annual temperatures varying from 14 to 20 °C. The precipitation is concentrated during spring and summer months, and decreases from NE to SW (from 1,000 to 400 mm)^38^. The ecoregion is dominated by a large number of fluvial-aeolic shallow lakes and low order rivers and streams that mostly belong to the Salado-Vallimanca basins^32^. Particularly, lakes are characterized by rounded contours and pan-shaped profiles. They are typically shallow, polymictic, eutrophic to hypertrophic, with highly variable water renewal time and salinity. Most of the surrounding land is devoted to agricultural practices^36^. This economic development directly affected shallow lakes, promoting shifts in many of them from clear regimes, characterized by the presence of submerged vegetation, to algal-dominated turbid states^68^.

24. Monte-Patagonian

24.2 Patagonian Tablelands

The Patagonian tablelands ecoregion (defined as “Patagonian plateau” by Quirós & Drago^32^, is a complex landscape of about 600,000 km^2^, located in Argentina (33.68°–54.52° S, 68.75°–66.35° W. It is delimited by the Colorado River to the North, the Atlantic Ocean to the East, the Andes to the West and parallel 54° to the South^69^. It is characterized by extreme conditions of cold and dry climate, with average maximum temperatures of 2.9 and 14.0 °C in winter and summer, respectively, and minimum temperatures can be below −19.0 °C in winter. The mean annual precipitation is ~300 mm. This ecoregion encompasses different types of water bodies, including reservoirs, permanent natural lakes and temporary ponds. Most water bodies are shallow lakes, typically ranging from mesotrophic to eutrophic. Climate conditions determine that small shallow lakes (i.e. less than 30 km^2^) usually remain frozen from early autumn throughout late spring, however during the ice-free period due to frequent strong winds, the water columns are continuously mixed, thus preventing the formation of stable thermoclines^70–72^.

## Data Records

**Fig. 2:**
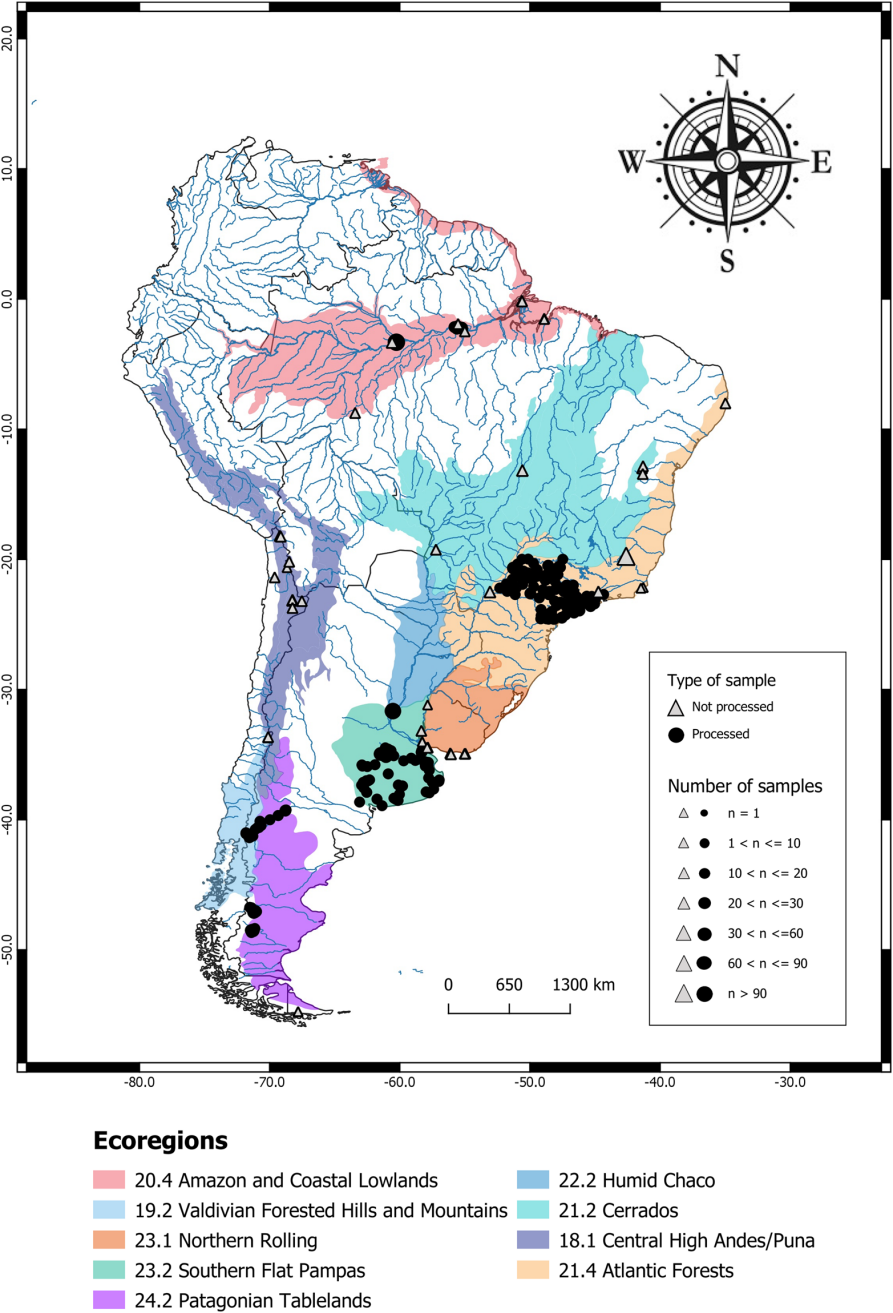
Sampling sites included in the **µSudAqua[db]** database by ecoregion. In color are highlighted the different ecoregions. The point size indicates the number of samples in the same sample site (e.g. time series). Triangles stand for samples sequenced with different primers from Herlemann & collaborators^21^. Those samples that constitute the **µSudAqua[db.sp]** database are indicated by circles.

The **µSudAqua[db]** covers 866 individual samples of continental waters from South America (Table 1, Fig. 2). It contains samples sequenced using 454, Ion Torrent and Illumina technologies, and targeting different hypervariable regions of the 16S rRNA gene. The raw samples files are freely available in the European Nucleotide Archive (ENA) database^73^. They can be downloaded using the Run Accession Number from the metadata file provided in Zenodo repository^22^.

The **µSudAqua[db.sp]** database is composed of 509 samples from 14 sequencing projects, representing ~60% of the data of the **µSudAqua[db]**. The ASVs’ information after sequencing processing using DADA2 pipeline (number of reads, nucleotide sequences and taxonomic classification) are also provided in different machine-reliable files at Zenodo^22^.

## Technical Validation

The technical validation was performed using the **µSudAqua[db.sp]**, that comprises the samples that were sequenced with the Illumina MiSeq technology, and targeted the V3-V4 regions of the 16S rRNA gene.

***µSudAqua[db.sp]*** *: Bacterial distribution among the ecoregions of South America*

In total, 509 samples and 116,687,584 reads were processed with DADA2. In order to exclude possible remaining sequencing errors or chimeras, we filtered ASVs with less than 50 reads in less than 3 samples. Thus, the final ASV table consisted of 502 samples, 25,334 ASVs and 42,188,085 reads, from: Amazon and Coastal Lowlands (96 samples), Atlantic Forests (67), Cerrados (86), Humid Chaco (59), from Southern Flat Pampas (127), Valdivian Forested Hills and Mountains (7) and, Patagonian Tablelands (67). The information of sequence processing and quality check of samples is summarized in Table 3.

**Fig. 3:**
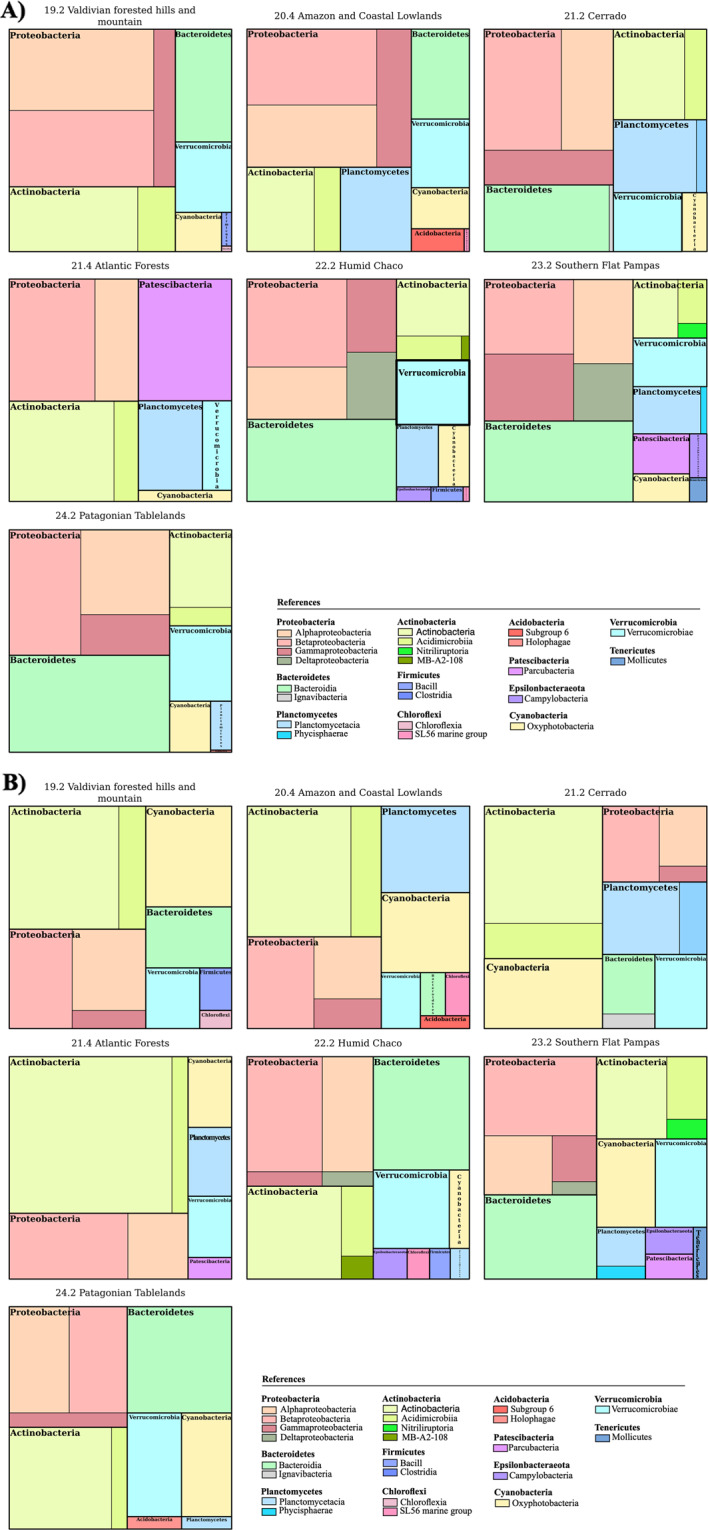
(**A**) Number of ASVs (richness) and (**B**) number of reads (relative abundance) of the major bacterial taxonomic groups that contribute with more than 1% of the total reads by ecoregion.

The **µSudAqua[db.sp]** database was mainly represented by Bacteria (24,279 ASVs, 97.7% reads) followed by chloroplasts (1,001 ASVs, 2.2% reads) and Archaea (54 ASVs, 0.03% reads). Within Bacteria, the reads were distributed in 6 principal taxonomic groups: Actinobacteria (33.4%), Proteobacteria (12.2% *Betaproteobacteria*, 7.6% *Alphaproteobacteria* and, 2.5% *Gammaproteobacteria*), Cyanobacteria (10.7%), Planctomycetes (9.5%), Bacteroidetes (8.6%) and Verrucomicrobia (6.7%). Bacteroidetes was the richest group (Bacteroidia, 3,359 ASVs), followed by Proteobacteria (*Betaproteobacteria*, 3,129 ASVs, *Alphaproteobacteria*, 2,133 ASVs, and *Gammaproteobacteria*, 1,648 ASVs) and Cyanobacteria (776 ASVs). The relative abundance and richness of each principal taxonomic group were notably different among the studied ecoregions (Fig. 3).

## Usage Notes

The links to download the raw fastq data from **µSudAqua[db] and µSudAqua[db.sp]** are in the **metadata** file accessible in Zenodo^22^. In addition, other files associated with the **µSudAqua[db.sp]** are available in the same repository: ASVs table (number of reads in each sample), taxonomy, nucleotide sequences in fasta format and ASVs table filtered with only Bacteria. Importantly, the database will grow as new samples and sequencing projects from the µSudAqua network appear. This information will be uploaded in the repository and the tables will be updated in future versions of the database. A bibliography revision and open call for new data submission will be performed once a year, and the database will be updated after data quality check, processing and integration.

The **µSudAqua[db]** and **µSudAqua[db.sp]** databases are the first to integrate information of microbial diversity from continental systems of South America, an important region that has been overlooked comparing to other regions and environments worldwide. These databases will open new avenues for studies on the temporal patterns and spatial distributions of microbial communities among the different ecoregions of South America. Besides, the integration of the curated data to meta-analysis of microbial communities from different ecosystems (comparison between South America and well-studied regions of the world), will be particularly important for exploring the novel microbial diversity, allowing to reveal regions with unknown organisms and functions, as well as hotspots of microbial biodiversity.

## Data Availability

The workflow included several custom-made R and python scripts, which are accessible GitHub (https://github.com/microsudaqua/usudaquadb).
